# Pendulous Fatty Tumour of Thigh

**Published:** 1885-03

**Authors:** Shirley Deakin

**Affiliations:** Surgeon, Bengal Service


### PENDULOUS FATTY TUMOUR OF THIGH.

By Shirley Deakin, F.R.C.S. Eng.; Surgeon, Bengal Service.

About seven years ago I removed a fatty tumour, very similar in appearance to that depicted in plate VII., p. 126 (1883) of the *Bristol Medico-Chirurgical Journal*. The patient, I believe, was a young man, a native of India, in good health, and the wound healed rapidly. When the scrotum was not held aside the tumour appeared to be a very pendulous scrotum at first sight. This is the only case that I remember to have seen in practice. I have notes of the case, but cannot lay my hands on them at present.

